# Phenotype-based management of coronary microvascular dysfunction

**DOI:** 10.1007/s12350-022-03000-w

**Published:** 2022-06-07

**Authors:** Daniel Tze Yee Ang, Colin Berry, Juan-Carlos Kaski

**Affiliations:** 1 British Heart Foundation Glasgow Cardiovascular Research Centre, University of Glasgow, Glasgow, United Kingdom; 2 Molecular and Clinical Sciences Research Institute, St George’s University of London, London, United Kingdom

**Keywords:** Microvascular dysfunction, myocardial blood flow, diagnostic and prognostic application, vasodilators, CAD, myocardial ischemia and infarction

## Abstract

40-70% of patients undergoing invasive coronary angiography with signs and symptoms of ischemia are found to have no obstructive coronary artery disease (INOCA). When this heterogeneous group undergo coronary function testing, approximately two-thirds have demonstrable coronary microvascular dysfunction (CMD), which is independently associated with adverse prognosis. There are four distinct phenotypes, or subgroups, each with unique pathophysiological mechanisms and responses to therapies. The clinical phenotypes are microvascular angina, vasospastic angina, mixed (microvascular and vasospastic), and non-cardiac symptoms (reclassification as non-INOCA). The Coronary Vasomotor Disorders International Study Group (COVADIS) have proposed standardized criteria for diagnosis. There is growing awareness of these conditions among clinicians and within guidelines. Testing for CMD can be done using invasive or non-invasive modalities. The CorMicA study advocates the concept of ‘functional angiography’ to guide stratified medical therapy. Therapies broadly fall into two categories: those that modulate cardiovascular risk and those to alleviate angina. Management should be tailored to the individual, with periodic reassessment for efficacy. Phenotype-based management is a worthy endeavor for both patients and clinicians, aligning with the concept of ‘precision medicine’ to improve prognosis, symptom burden, and quality of life. Here, we present a contemporary approach to the phenotype-based management of patients with INOCA.

**Supplementary Information:**

The online version contains supplementary material available at 10.1007/s12350-022-03000-w.

## Background

Angina pectoris affects approximately 112 million people globally.^1^ Approximately 40-70% of patients undergoing invasive coronary angiography with signs and symptoms of ischemia are found to have no obstructive coronary artery disease (INOCA).^2-4^ Of these, close to two-thirds have demonstrable coronary microvascular dysfunction (CMD) when investigated with coronary function tests.^5-7^ While CMD may co-exist with epicardial atherosclerosis, there is growing evidence that this condition is independently associated with persistent angina symptoms, impaired quality of life and increased risk of death, myocardial infarction and stroke.^4,7-9^

The true prevalence of CMD in the community is difficult to estimate. This is due to a myriad of factors, including limited testing in clinical practice, availability of different testing modalities in each center, varying test sensitivities and specificities, and research populations representing a select group of patients. The last factor is particularly relevant in cohorts undergoing invasive management in the cardiac catheterization laboratory. For instance in the ISCHEMIA trial, 20% of patients with moderate to severe ischemia were found to have non-obstructed coronaries on initial CT coronary angiography, and did not proceed to randomization.^10,11^

Patients with INOCA are a heterogeneous group, with varying pathophysiological mechanisms underpinning the supply-demand mismatch of coronary artery blood flow. These distinct subgroups, or phenotypes, are therefore responsive to different therapies. Diagnostic criteria and linked therapy for these conditions are now acknowledged in expert consensus and contemporary guideline documents.^7,12,13^

While the initial presenting symptoms may be similar, it is important to manage these patients according to phenotype, in order that the best outcomes are achieved. Indeed, the CorMicA pilot study demonstrates that stratified medical therapy, as guided by an interventional diagnostic procedure (IDP) at the time of invasive coronary angiography, improves angina symptom burden and quality of life in patients with INOCA.^5,14^

Here, we present a contemporary approach to the management of patients with INOCA, focusing on the main clinical phenotypes, specifically: microvascular angina, vasospastic angina, mixed (microvascular and vasospastic), and non-cardiac symptoms.

## Phenotypes and endotypes defined

Phenotype and endotype have distinct meanings. Phenotype reflects observable clinical characteristics such as eye color, blood type, body weight, and blood pressure. Phenotype is determined by genetic and environmental characteristics. Phenotype may reflect a clinical condition, such as angina, heart failure, or stroke. Endotype is subtype of a condition, which is characterized by a distinct functional or pathophysiological mechanism.^15^

Patients with CMD present with a wide spectrum of signs and symptoms. Many, including dyspnea, are considered atypical and often misdiagnosed as non-cardiac in origin. This is particularly true for women, who are less likely to have obstructive coronary disease and more likely to have CMD.^16^ The first step in managing INOCA is to accurately discriminate between the four distinct phenotypes, summarized below.

### Microvascular angina (MVA)

MVA may be due to a structural and/or functional problem of the coronary microcirculation. In other words, the endotypes are structural MVA and functional MVA. Structural MVA is caused by remodeling of the microvascular circulation and/or extracellular matrix. Functional MVA reflects a vasomotion disorder of the coronary arterioles. Functional MVA may be due to impaired vasodilator reserve, i.e., reduced CFR, and/or microvascular spasm. This then results in fixed or dynamic flow reduction, respectively.^17^ These mechanisms may co-exist. Remodeled arterioles may also be more reactive to vasoconstricting stimuli. These mechanisms serve as potential targets for therapy.

Historically, MVA could be considered a more nebulous diagnosis with wide variation in practice. In response, the Coronary Vasomotor Disorders International Study (COVADIS) Group have proposed standardized diagnostic criteria for MVA to improve patient care^17^: (1) anginal symptoms, (2) absence of obstructive coronary artery disease, (3) objective evidence of myocardial ischemia, and (4) evidence of coronary microvascular dysfunction. ‘Definite MVA’ is established if all 4 criteria are met, or ‘Suspected MVA’ if only 3 are present.

### Vasospastic angina (VSA)

Prinzmetal et al. first described focal epicardial vasospasm as ‘variant angina’.^18^ The main pathophysiological process underpinning this condition is coronary circulation hypersensitivity to vasoconstrictor stimuli.^19,20^ This includes spasm occurring both spontaneously, as well as during pharmacological reactivity testing. VSA typically occurs at rest, particularly during the night or early morning hours, is associated with transient ST segment elevation and can be precipitated by stress, allergic reactions, hyperventilation, and ergot derivatives or more rarely, be induced by exertion. Acetylcholine and ergonovine are triggers of spasm currently used as diagnostic tests in the catheterization laboratory.^21-24^

Coronary vasospasm may present in guises other than “variant angina”, with ST segment depression or T wave changes, instead of ST segment elevation and rest and effort-induced angina. Vasospasm affecting the microvessels, reported as microvascular spasm, is a relatively frequent occurrence associated with ischemic ECG changes, myocardial perfusion abnormalities and elevations of high sensitivity troponin.^25-27^ Provocative tests used for the diagnosis of epicardial coronary artery spasm are also able to identify microvascular spasm.

Epicardial spasm is confirmed when there is dynamic reduction of >90% in coronary luminal diameter, along with reproduction of symptoms and ischemic ECG changes.^7,28^ Microvascular spasm is instead a pathology of microcirculatory arteriolar constriction, presenting with increased Thrombolysis in Myocardial Infarction (TIMI) frame count (cutoff > 27, at angiographic setting of 30 frames/second), or reduction in antegrade coronary flow (TIMI flow), reminiscent of the ‘no reflow’ phenomenon.

### Mixed (microvascular and vasospastic)

MVA and VSA may co-exist, reflecting both structural and vasomotor abnormalities. These patients may have worse overall quality of life compared with isolated MVA.^29^

### Non-cardiac symptoms

This diagnosis of exclusion should only be established after systematic assessment of coronary microvascular function, for instance if diagnostic guidewire and pharmacological reactivity tests are normal.

## Diagnostic testing

Contemporary European and North American guidelines have provided a IIa “should be considered” recommendation for the assessment of CMD.^12,13^ Both invasive and non-invasive options are available, with the objective of improving patient wellbeing and quality of life by stratifying and treating patients by phenotype. Invasive testing involves instrumentation of a single coronary artery at a time, and sequential assessments of other coronary arteries may be needed to rule out heterogeneous test results across the coronary circulation.^30^ Multivessel testing only becomes relevant in the event of initially normal test results in a symptomatic patient.

The main drawback of non-invasive modalities is the lack of pharmacological reactivity testing. Most non-invasive methods also require correlation with anatomical imaging to first exclude obstructive epicardial coronary disease. Modalities like PET and MRI also form assessments based only on estimation of flow at rest vs during hyperemia, without the option of estimating pressure and/or resistance in the coronary microcirculation.

An alternative empirical approach by some clinicians is to forego CMD testing and instead proceed directly to a trial of therapy in all patients. Proponents of this approach cite reduced health resource utilization initially. However, clinical follow-up will be needed, and this may be prolonged and less efficient given the lack of diagnostic information on disease mechanisms. Conversely, stratified therapy according to phenotype is more in keeping with the concept of ‘Precision Medicine’, allowing the right treatment to be administered to the right patient, at the right time.^15^ It allows personalization of therapy according to the underlying disease mechanism, or cessation of unnecessary medication in the case of non-cardiac symptoms.

### Invasive testing

**Figure 1:**
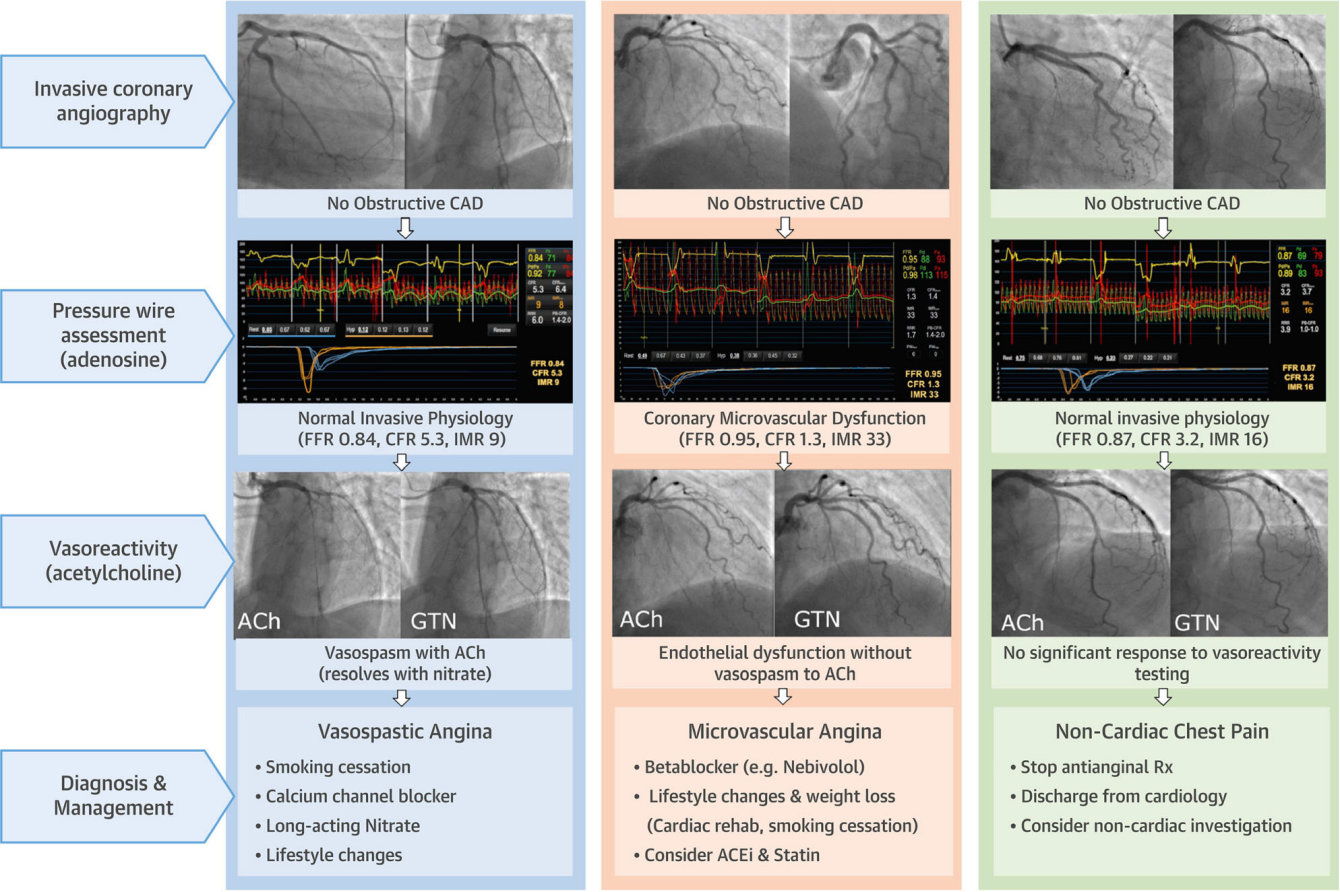
Stratified medical therapy guided by the interventional diagnostic procedure (IDP) in patients with INOCA. *CAD*, coronary artery disease; *FFR*, fractional flow reserve; *CFR*, coronary flow reserve; *IMR*, index of microvascular resistance; *ACh*, acetylcholine; *GTN*, glyceryl trinitrate; *Rx*, therapy. Reprinted from the Journal of the American College of Cardiology, Vol 72(23 Pt A), Ford TJ, Stanley B, Good R, Rocchiccioli P, McEntegart M, Watkins S, Eteiba H, Shaukat A, Lindsay M, Robertson K, Hood S, McGeoch R, McDade R, Yii E, Sidik N, McCartney P, Corcoran D, Collison D, Rush C, McConnachie A, Touyz RM, Oldroyd KG, Berry C. Stratified Medical Therapy Using Invasive Coronary Function Testing in Angina: The CorMicA Trial, Page 2843, Copyright (2018), with permission from Elsevier

An interventional diagnostic procedure (IDP) may be performed at the time of invasive coronary angiography in selected patients. The IDP comprises two components: a diagnostic guidewire test and a pharmacological coronary reactivity test.^31^ Figure 1 provides a summary diagram with example results from the CorMicA study.

Briefly, the diagnostic guidewire utilizes either combined pressure/temperature or pressure/ultrasound technology to estimate flow in the coronary microvasculature. The test seeks to assess the vasodilator capacity (coronary flow reserve [CFR], CFR < 2.0 abnormal, 2.0-2.5 ‘grey zone’) and resistance (index of microvascular resistance [IMR], IMR ≥ 25 abnormal; hyperemic microvascular resistance [HMR], HMR ≥ 2.5 mmHg/cm per second abnormal) of the microvasculature circulation.

The pharmacological provocation test involves intracoronary infusion of a vasoactive substance, commonly acetylcholine or ergonovine, to assess the vasodilator potential and propensity to vasospasm of the coronary circulation. Acetylcholine testing revealed an 33-45% incidence of epicardial spasm and 24-55% incidence of microvascular spasm, when undertaken in patients with INOCA^21,22^ Provocation testing is of particular merit in patients with angina at rest and in those who do not respond to conventional therapy.

A systematic review of 9,444 patients comparing acetylcholine with ergonovine as an intracoronary agent demonstrated a good safety profile.^24^ The incidence of major complications (VT, VF, prolonged refractory coronary spasm, MI, coronary dissection) was .8%, with no fatalities. Minor complications were quoted at 4.7%, predominantly transient AV nodal block, atrial fibrillation, and transient hypotension. Acetylcholine was thought to be slightly more sensitive as a diagnostic agent, but at the cost of minor increase in complication rates. Ultimately, these rates are low and comparable to those quoted for standard diagnostic angiography. Subsequent studies have suggested even greater safety if utilizing slower infusions in a stepwise manner.^21,32^

### Non-invasive testing

**Figure 2:**
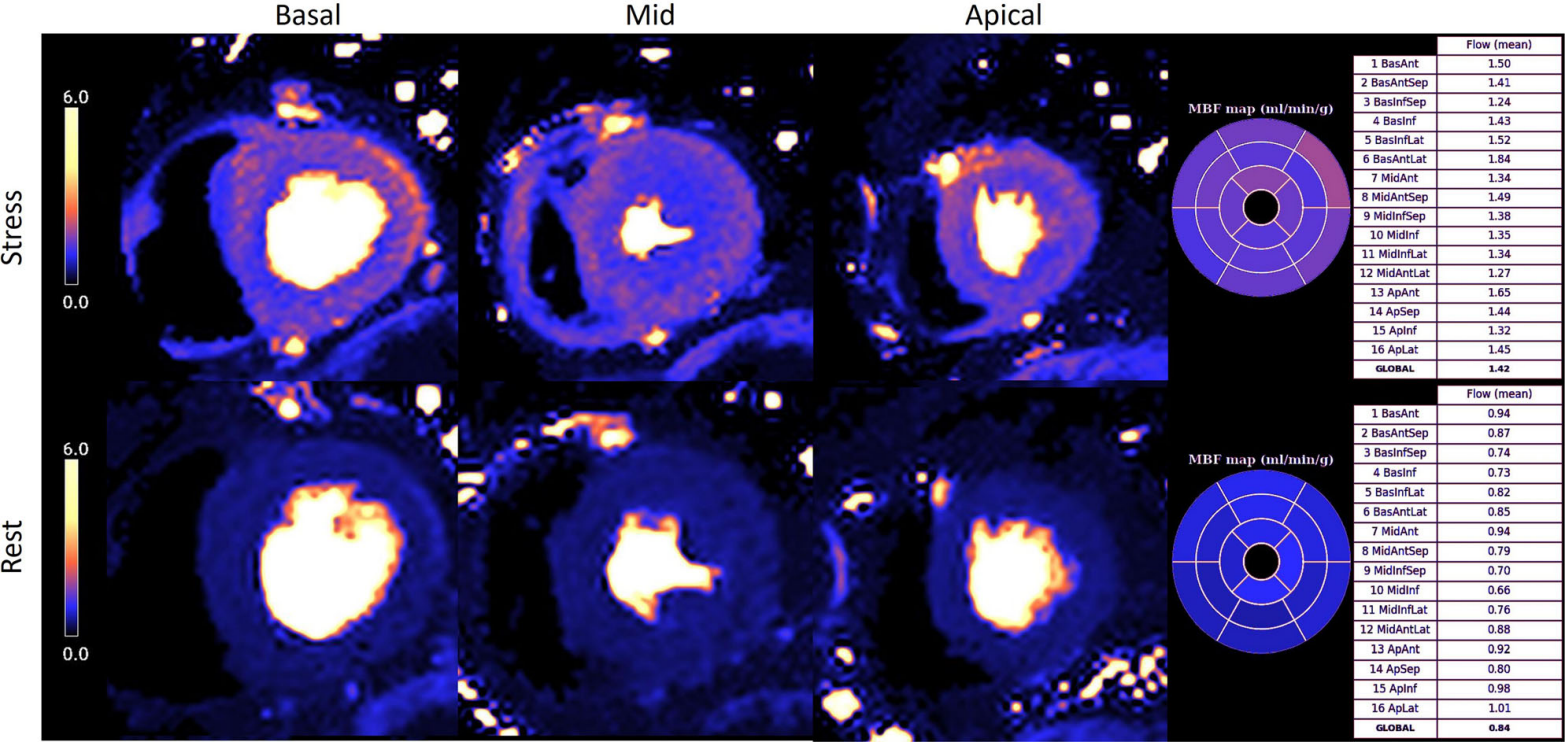
Example of abnormal perfusion scanning results on cardiac MRI. The stress perfusion images in the top row demonstrate a circumferential subendocardial perfusion defect when compared with those at rest in the lower row. The calculated global stress myocardial blood flow (MBF) is low at 1.42ml/g/min (abnormal < 2.25). Myocardial perfusion reserve (MPR) is also reduced at 1.69 (abnormal < 2.2). These results are indicative of CMD

The most widely utilized modalities are myocardial perfusion imaging using PET^33^ or cardiac MRI.^34^ Reduction in myocardial perfusion reserve (MPR) calculated from both modalities have been shown to be an independent predictor of major adverse cardiovascular events.^35,36^ A major advantage of cardiac MRI is its simultaneous assessment of cardiac structure, function, and tissue characterization. An example of abnormal myocardial perfusion results in an INOCA patient with CMD is shown in Fig. 2.

Another option is the use of transthoracic Doppler echocardiography to calculate coronary flow velocity ratio (CFVR) in the left anterior descending (LAD) coronary artery.^37^ Although inexpensive, this technique is limited by operator dependency and patient acoustic windows.

## Management according to phenotype

### General approach and considerations

As highlighted by Boden et al., optimal medical therapy is a constantly shifting target that should be reflective of the current best medical treatments and strategies.^38^ The goals of therapy are dual: (1) to improve clinical outcomes through the reduction of adverse cardiovascular events, and (2) to improve symptom burden and quality of life. As with many medical conditions, treatments for CMD broadly fall into two categories: lifestyle management and medication.

A patient-centered approach is key. Management decisions will vary among patients according to personal expectations, baseline health and function, mechanisms of angina in the individual patient, the co-existence of obstructive disease, MVA and vasospasm, co-morbidities, and willingness to persevere with up titration of medication. The best management plans are therefore joint decisions between the patient, their family, and clinician, tailored according to their individual needs and objectives.

### Cardiovascular risk factor control

COURAGE and BARI-2D have defined optimal medical therapy for the prevention of cardiovascular events as a combination of antiplatelet therapy, lipid lowering therapy (in particular statins), and inhibitors of the renin-angiotensin-aldosterone system.^39,40^ More recently, the ISCHEMIA trial also considered as main-line therapy PCSK9-inhibitors for more potent lipid control, direct oral anticoagulants (DOACs) for reduction of residual thrombotic risk, and SGLT2-inhibitors for improved diabetes control.

Smoking cessation and avoidance of environmental smoking exposure should be strongly encouraged. Risk factor therapy should be goal-directed to achieve target blood pressure, lipid, BMI and glycemic control.^7,12^ A healthy diet and exercise training, including engagement with cardiac rehabilitation if necessary, should be encouraged.^41^ These same targets also apply to patients with a diagnosis of non-cardiac symptoms if atherosclerotic plaque is identified during angiography.

WARRIOR is a phase 3, randomized, controlled clinical trial investigating whether intensive medical therapy and optimal control of cardiovascular risk factors can improve health outcomes in women with INOCA.^42^

### Phenotype-specific angina therapy

**Figure 3:**
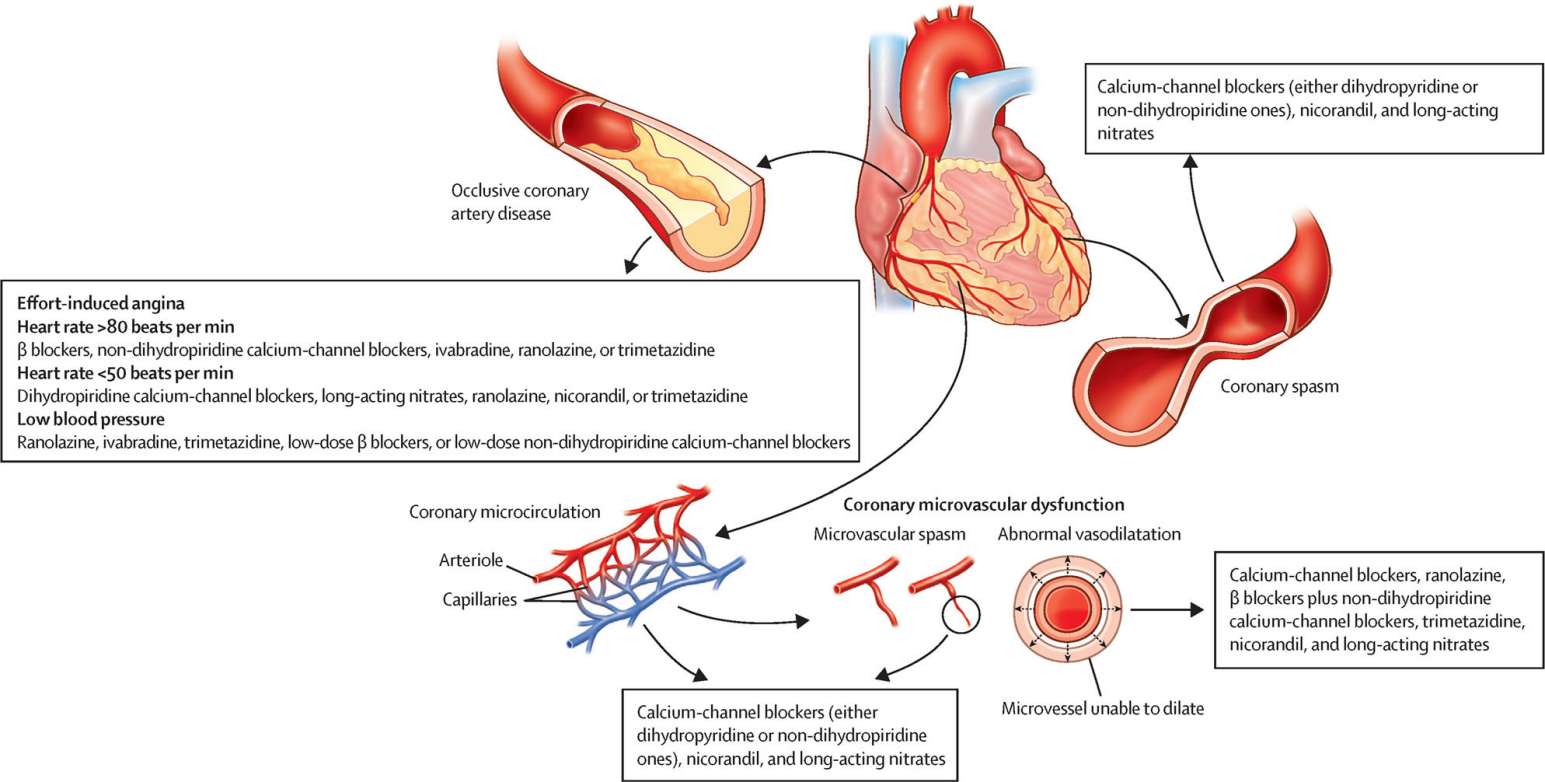
Management of angina according to the underlying disease mechanisms. Reprinted from The Lancet, Vol 399, Boden WE, Kaski JC, Al-Lamee R, Weintraub WS. What constitutes an appropriate empirical trial of antianginal therapy in patients with stable angina before referral for revascularisation?, Page 693, copyright (2022), with permission from Elsevier

The choice of antianginal agent should be decided according to the predominant mechanism of angina in that patient, i.e., abnormal vasodilation, coronary spasm, or suboptimal hemodynamic control. Diagnosis-specific therapies are established by consensus documents.^7^ A key tenet in the management of CMD is the periodic reassessment of symptoms, ideally monthly, to allow adjustment of medication doses, assessment for side-effects or addition of other agents. The approach for each phenotype is summarized in Fig. 3.

#### Microvascular angina

Although obviously a lot less accurate than the information provided by invasive coronary physiology studies and non-invasive tests such as PET or cardiac MRI perfusion scans, the clinical presentation with predominantly effort-induced angina, rest-angina suggestive of vasospasm or “mixed” effort and rest angina, helps clinicians to at least suspect the prevailing mechanism of angina in a given patient and identify suitable initial antianginal therapies. First line therapy in effort-induced MVA entails a trial of beta-blockers (selective beta-blockers, or combined alpha/beta-blockers) or calcium channel antagonists (dihydropyridine and/or non-dihydropyridine). Long-acting nitrates should be avoided in general, as they are frequently ineffective and may aggravate symptoms due to a ‘steal effect’.^43^ However, up to 50% of MVA patients find short-acting sublingual nitrates, oral nitrates or skin GTN patches helpful.^22^ Nicorandil may be helpful in cases of vasospastic MVA, as it is in patients with variant angina. These therapies aid with vasodilatation as well as reduction in myocardial oxygen consumption.^7^

Renin-angiotensin system blockade is recommended.^44^ ACE inhibitors have been demonstrated to improve hyperemic myocardial blood flow, CFR and angina burden in patients with MVA.^45^ Statins may have additional benefit in MVA, over and above standard lipid control and plaque modification. Studies suggest anti-inflammatory properties, increase in endothelial nitric oxide release and inhibition of the rho kinase pathway in vascular smooth muscle cells, which leads to vasodilatation.^46^

Ranolazine is mentioned in guidance documents,^7,12^ with potentially better response in patients with low CFR.^47^

#### Vasospastic angina

Patients with epicardial or microvascular spasm should undergo initial therapy with calcium channel blockade. This encourages vascular smooth muscle relaxation, reduces propensity toward spasm, and potentially reduces myocardial oxygen demand. High doses and/or a combination of non-dihydropyridine (verapamil or diltiazem) with dihydropyridine (amlodipine) calcium blockers may be required. In contrast to MVA, long-acting nitrates are useful adjuncts in epicardial spasm, while beta-blockers are avoided. Nicorandil is also an option.

#### Mixed (microvascular and vasospastic)

The combination of treatments in this group reflects the mixed pathologies described above. Calcium channel antagonists (dihydropyridine and/or non-dihydropyridine) are considered first line, followed by nicorandil and/or third line agents such as ranolazine or trimetazidine.

#### Non-cardiac symptoms

When other CMD phenotypes have been excluded, alternative etiologies for the patient’s symptoms should be sought. Known cardiac mimics include gastroesophageal, lung, musculoskeletal, and nerve disorders. Antianginal therapy should be discontinued, but cardiovascular risk control should still be pursued if atherosclerosis is detected.

## Future developments

While the CorMicA pilot study was successful in demonstrating the benefit of phenotype-based management and guideline-directed optimal medical management as guided by the IDP, larger scale, international studies are required to bolster practice guidelines. To this end, the international iCorMicA (NCT04674449) and WARRIOR trials^42^ are currently recruiting.

Most standard anti-anginal therapies were designed with a focus on obstructive epicardial disease, with only limited efficacy in CMD.^48^ Upcoming CMD-specific therapy trials include the PRIZE study (NCT04097314) which investigates the use of an oral endothelin A receptor antagonist (Zibotentan), and coronary sinus device therapies (NCT02710435; NCT03625869).

## Conclusion

Patients with CMD remain a diagnostic and therapeutic challenge in clinical practice. There is growing evidence that a phenotype-specific approach to management improves patient outcomes. This heterogeneous group of patients should undergo appropriate testing to discern the underlying diagnosis, with therapy then tailored on an individualized basis. Treatment involves intensive lifestyle and medication intervention to achieve control of cardiovascular risk factors, with frequent reassessment for efficacy. Such efforts are a worthy investment for both patients and clinicians to improve prognosis, symptom burden, and quality of life.

## Supplementary Information

Below is the link to the electronic supplementary material.

Supplementary file1 (PPTX 871 kb)

## Abbreviations

CFR: Coronary flow reserve
CMD: Coronary microvascular dysfunction
HMR: Hyperemic microvascular resistance
IDP: Interventional diagnostic procedure
IMR: Index of microvascular resistance
INOCA: Ischemia with no obstructive coronary artery disease
MVA: Microvascular angina
RRR: Resistive reserve ratio
TIMI: Thrombolysis in myocardial infarction
VSA: Vasospastic angina
