# Supplementary material for: Phenotype-based management of coronary microvascular dysfunction
Source: J Nucl Cardiol. 2022 Jun 7;29(6):3332–40. doi: 10.1007/s12350-022-03000-w (PMC9834338; doi:10.1007/s12350-022-03000-w)
Supplement: Supplementary file 1 — Supplementary file1 (PPTX 871 kb) [file 12350_2022_3000_MOESM1_ESM.pptx]

## Slide 1
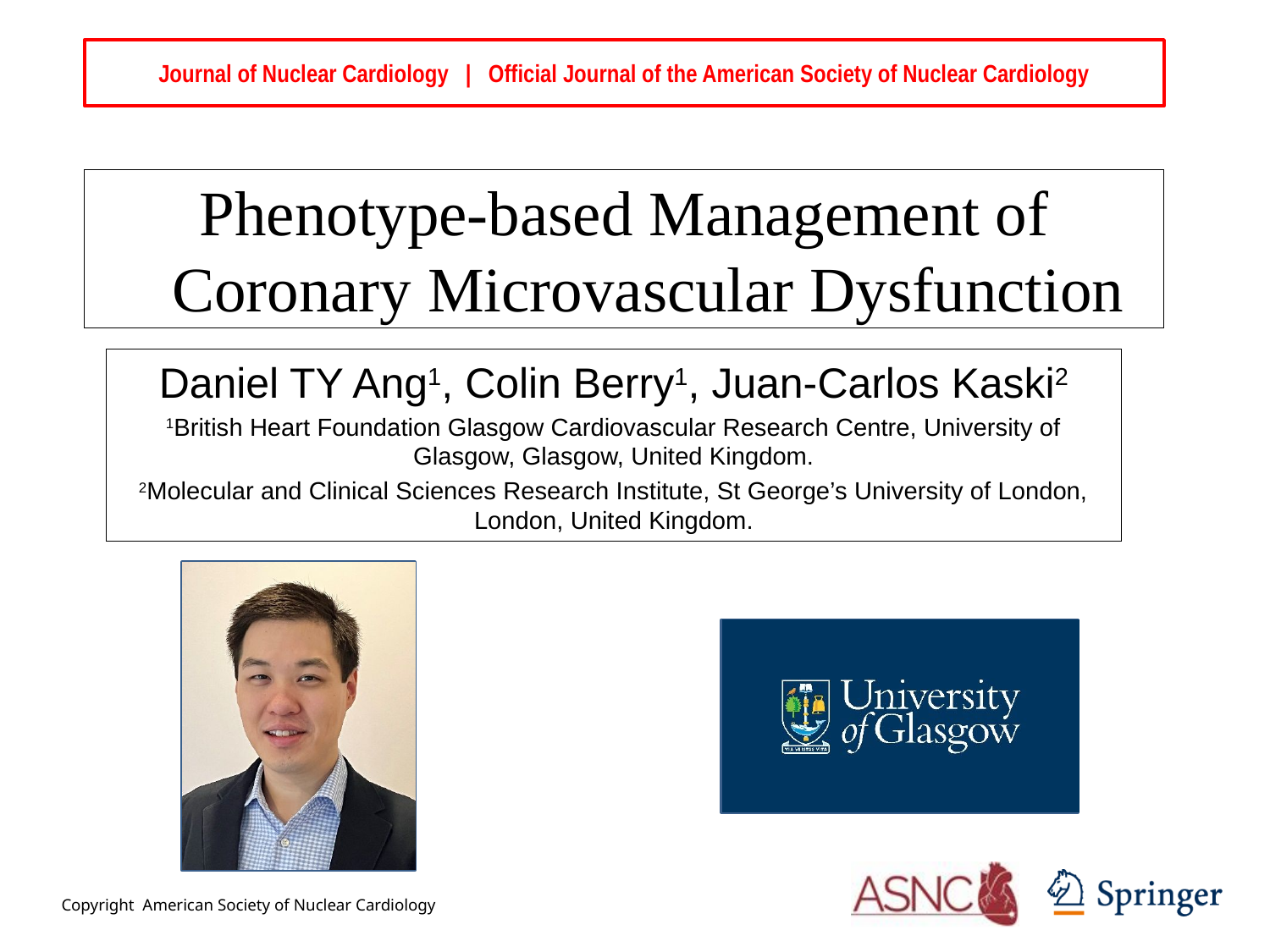

Journal of Nuclear Cardiology | Official Journal of the American Society of Nuclear Cardiology
# Phenotype-based Management of Coronary Microvascular Dysfunction
Daniel TY Ang1, Colin Berry1, Juan-Carlos Kaski2
1British Heart Foundation Glasgow Cardiovascular Research Centre, University of Glasgow, Glasgow, United Kingdom.
2Molecular and Clinical Sciences Research Institute, St George’s University of London, London, United Kingdom.
Copyright American Society of Nuclear Cardiology

## Slide 2
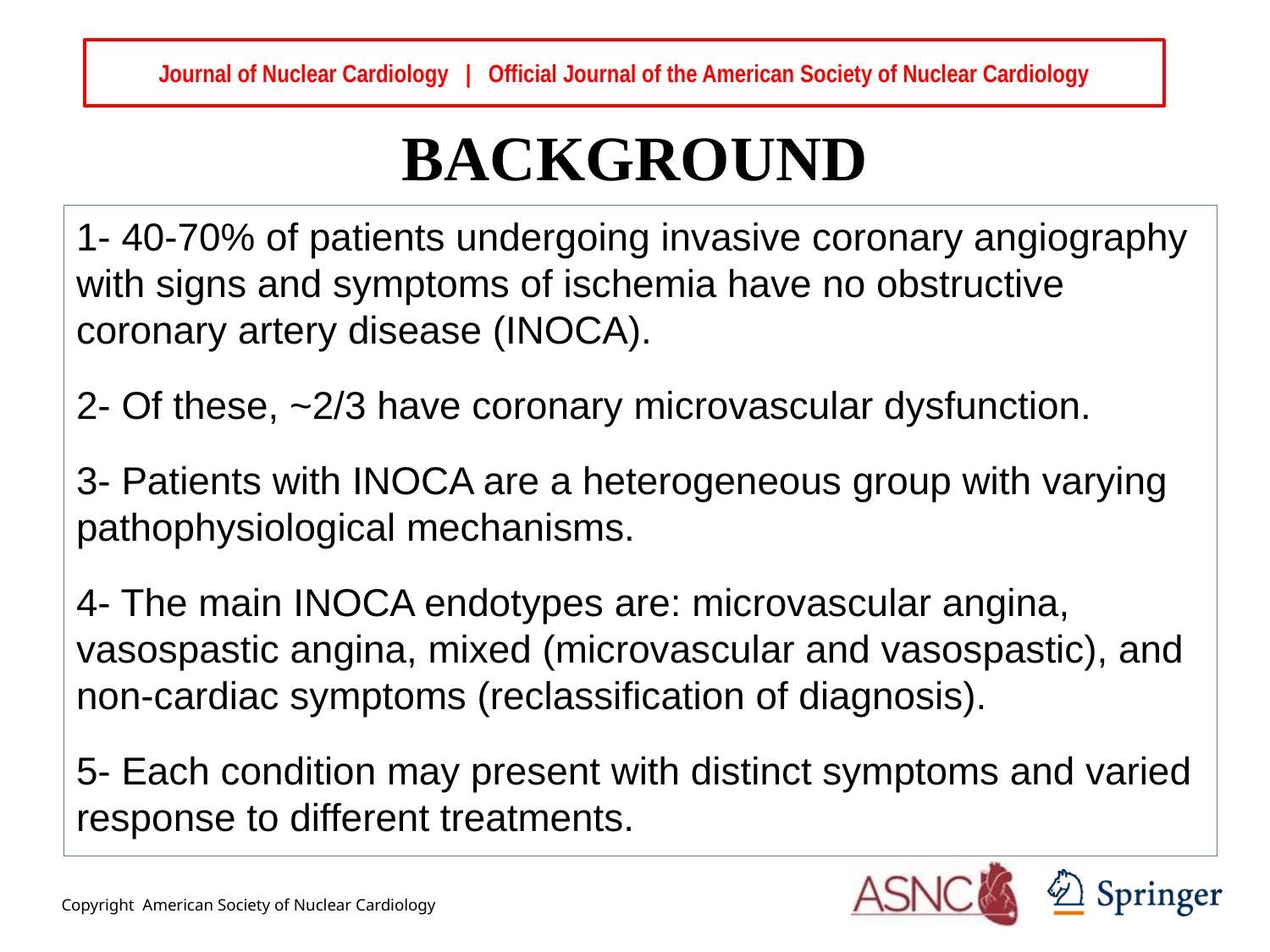

Journal of Nuclear Cardiology | Official Journal of the American Society of Nuclear Cardiology
# BACKGROUND
1- 40-70% of patients undergoing invasive coronary angiography with signs and symptoms of ischemia have no obstructive coronary artery disease (INOCA).
2- Of these, ~2/3 have coronary microvascular dysfunction.
3- Patients with INOCA are a heterogeneous group with varying pathophysiological mechanisms.
4- The main INOCA endotypes are: microvascular angina, vasospastic angina, mixed (microvascular and vasospastic), and non-cardiac symptoms (reclassification of diagnosis).
5- Each condition may present with distinct symptoms and varied response to different treatments.
Copyright American Society of Nuclear Cardiology

## Slide 3
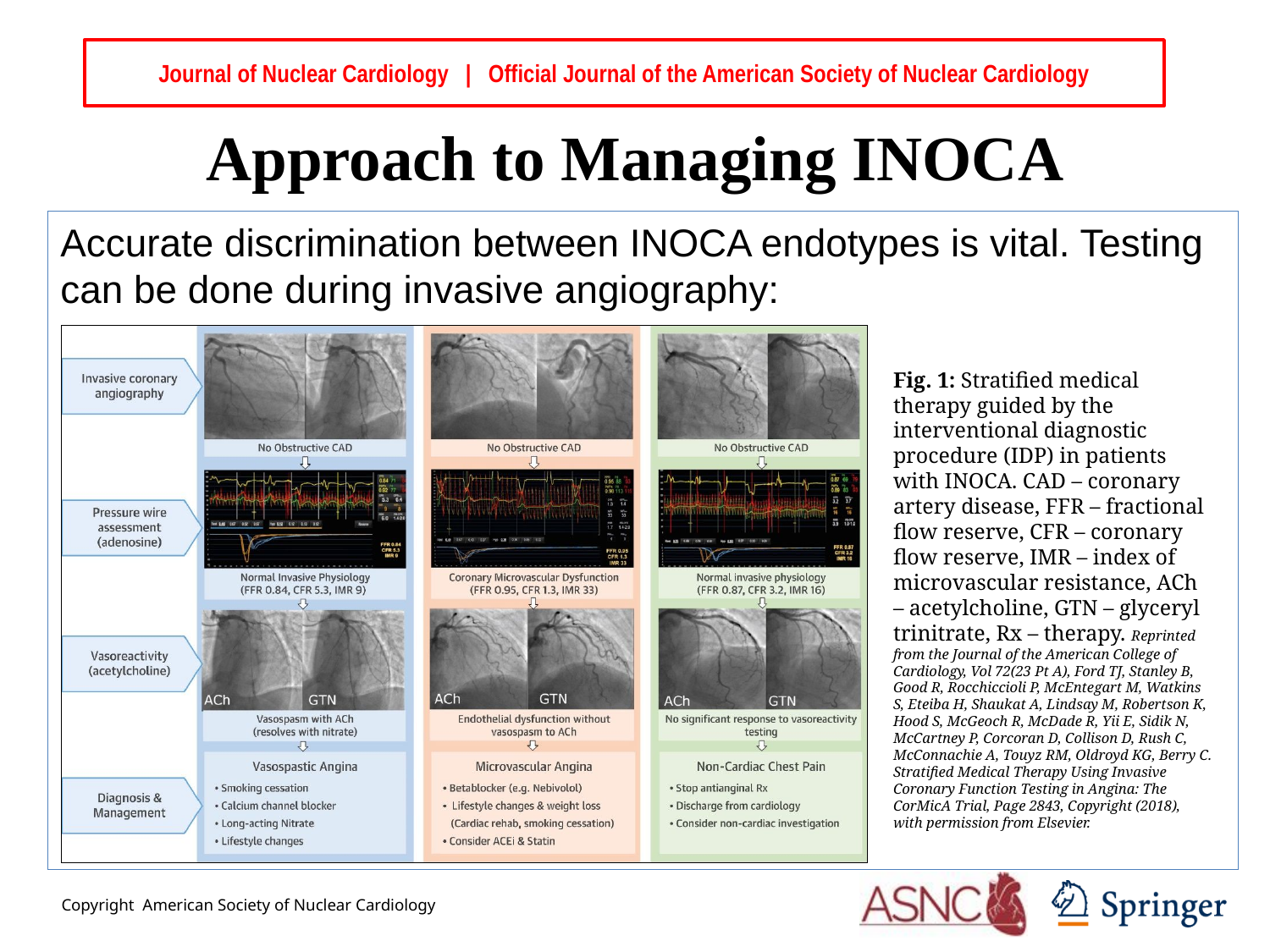

Journal of Nuclear Cardiology | Official Journal of the American Society of Nuclear Cardiology
# Approach to Managing INOCA
Accurate discrimination between INOCA endotypes is vital. Testing can be done during invasive angiography:
Fig. 1: Stratified medical therapy guided by the interventional diagnostic procedure (IDP) in patients with INOCA. CAD – coronary artery disease, FFR – fractional flow reserve, CFR – coronary flow reserve, IMR – index of microvascular resistance, ACh – acetylcholine, GTN – glyceryl trinitrate, Rx – therapy. Reprinted from the Journal of the American College of Cardiology, Vol 72(23 Pt A), Ford TJ, Stanley B, Good R, Rocchiccioli P, McEntegart M, Watkins S, Eteiba H, Shaukat A, Lindsay M, Robertson K, Hood S, McGeoch R, McDade R, Yii E, Sidik N, McCartney P, Corcoran D, Collison D, Rush C, McConnachie A, Touyz RM, Oldroyd KG, Berry C. Stratified Medical Therapy Using Invasive Coronary Function Testing in Angina: The CorMicA Trial, Page 2843, Copyright (2018), with permission from Elsevier.
Copyright American Society of Nuclear Cardiology

## Slide 4
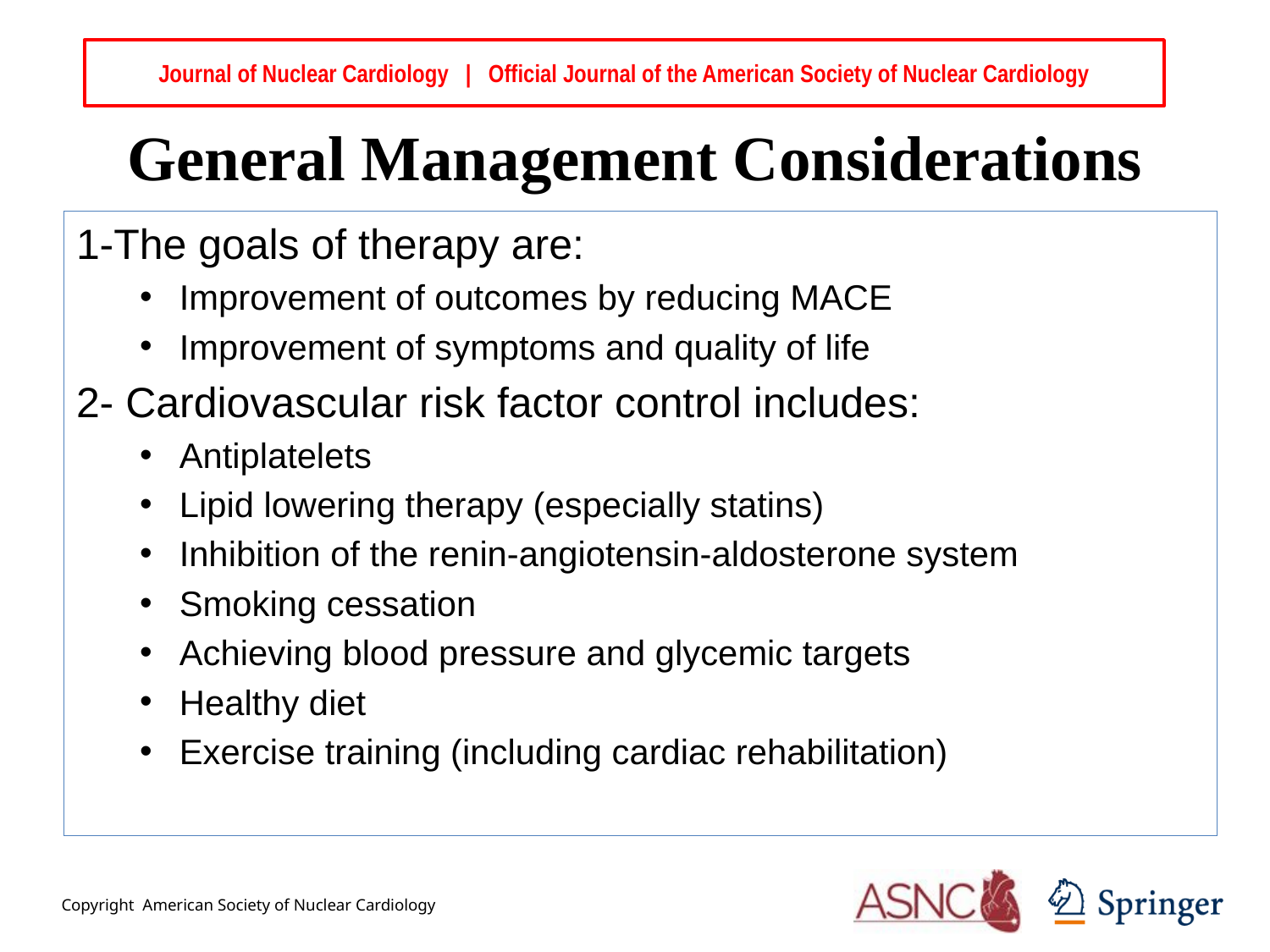

Journal of Nuclear Cardiology | Official Journal of the American Society of Nuclear Cardiology
# General Management Considerations
1-The goals of therapy are:
Improvement of outcomes by reducing MACE
Improvement of symptoms and quality of life
2- Cardiovascular risk factor control includes:
Antiplatelets
Lipid lowering therapy (especially statins)
Inhibition of the renin-angiotensin-aldosterone system
Smoking cessation
Achieving blood pressure and glycemic targets
Healthy diet
Exercise training (including cardiac rehabilitation)
Copyright American Society of Nuclear Cardiology

## Slide 5
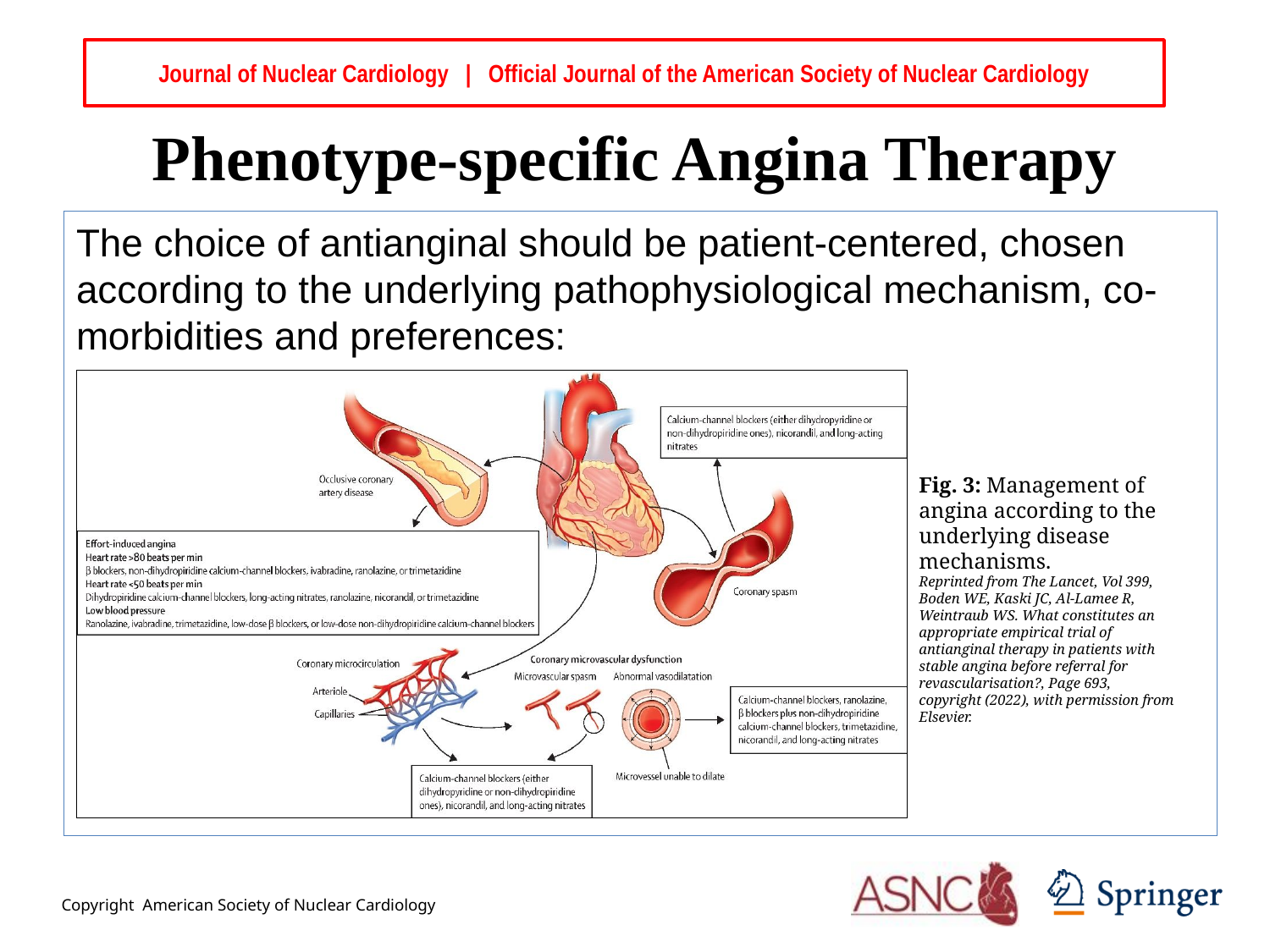

Journal of Nuclear Cardiology | Official Journal of the American Society of Nuclear Cardiology
# Phenotype-specific Angina Therapy
The choice of antianginal should be patient-centered, chosen according to the underlying pathophysiological mechanism, co-morbidities and preferences:
Fig. 3: Management of angina according to the underlying disease mechanisms.
Reprinted from The Lancet, Vol 399, Boden WE, Kaski JC, Al-Lamee R, Weintraub WS. What constitutes an appropriate empirical trial of antianginal therapy in patients with stable angina before referral for revascularisation?, Page 693, copyright (2022), with permission from Elsevier.
Copyright American Society of Nuclear Cardiology

## Slide 6
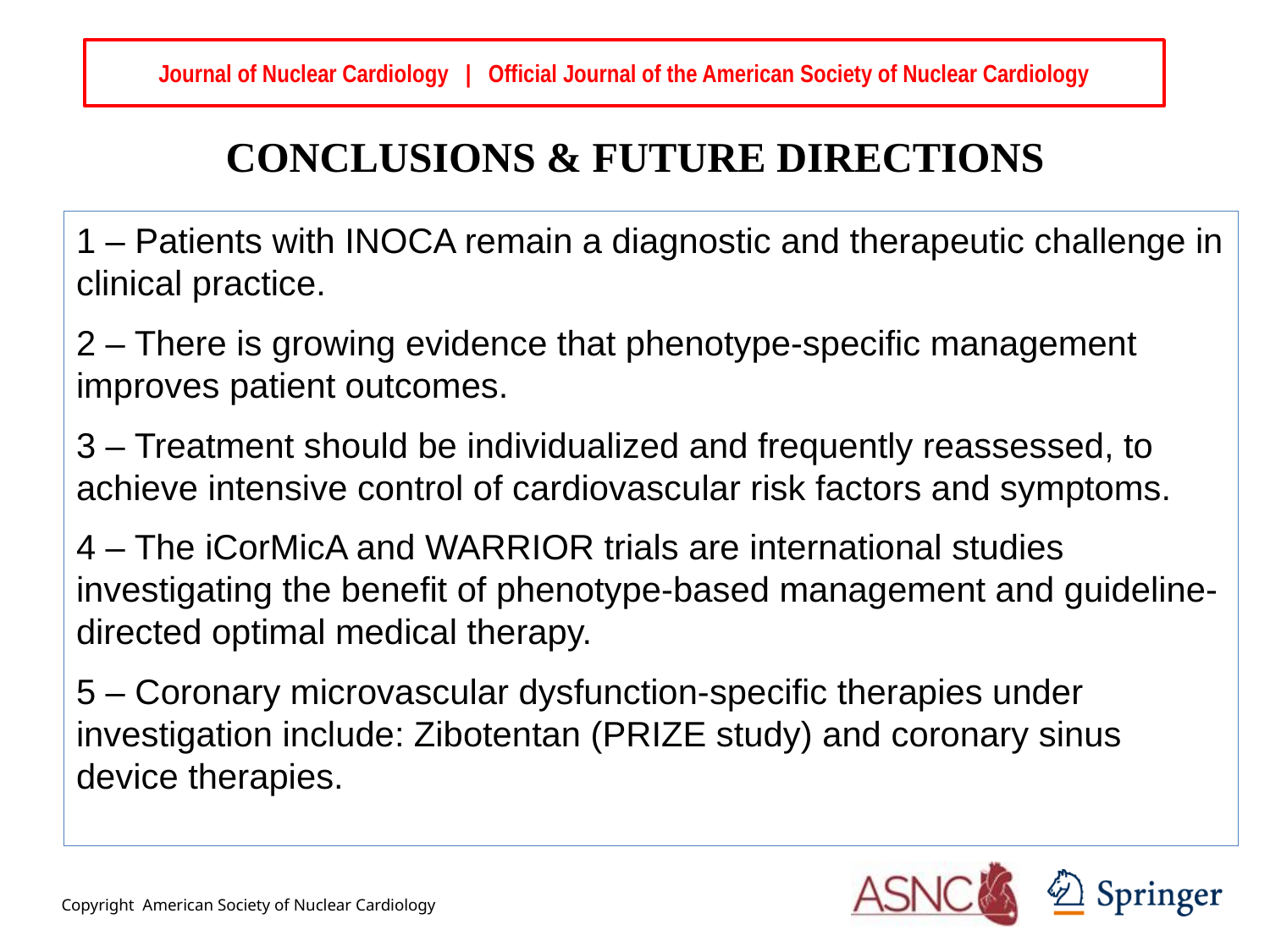

Journal of Nuclear Cardiology | Official Journal of the American Society of Nuclear Cardiology
# CONCLUSIONS & FUTURE DIRECTIONS
1 – Patients with INOCA remain a diagnostic and therapeutic challenge in clinical practice.
2 – There is growing evidence that phenotype-specific management improves patient outcomes.
3 – Treatment should be individualized and frequently reassessed, to achieve intensive control of cardiovascular risk factors and symptoms.
4 – The iCorMicA and WARRIOR trials are international studies investigating the benefit of phenotype-based management and guideline-directed optimal medical therapy.
5 – Coronary microvascular dysfunction-specific therapies under investigation include: Zibotentan (PRIZE study) and coronary sinus device therapies.
Copyright American Society of Nuclear Cardiology
